# Implementation status and quality improvement strategies of the record-filing system for drug clinical trial institutions in Sichuan Province

**DOI:** 10.3389/fmed.2026.1755380

**Published:** 2026-03-04

**Authors:** Shangyuan Qin, Houfeng Zhou

**Affiliations:** Department of Pharmacy, Chengdu Fifth People’s Hospital, Chengdu, China

**Keywords:** drug clinical trial institution, quality management, record-filing system, regulation, Sichuan Province

## Abstract

**Objective:**

This study systematically analyzes the implementation status and existing problems of the record-filing system for drug clinical trial institutions, proposes quality improvement strategies based on empirical data from Sichuan Province, and provides references for the optimization of the national record-filing system.

**Methods:**

Based on the National Drug Clinical Trial Institution Record-Filing Management Information Platform and publicly available data, descriptive statistics, trend analysis, and concentration analysis (Herfindahl–Hirschman Index, HHI) are adopted. Data cleaning was performed to exclude duplicate records and entries with missing key information (e.g., institution location, specialty registration). Key metrics were clearly defined: ① Institution coverage rate = (Number of registered institutions in the region/Total number of tertiary hospitals in the region) × 100%; ② Regional concentration degree was measured by HHI (calculated as the square of the proportion of institutions in each prefecture-level city to the provincial total); ③ Comparative indices included inter-provincial quantity ranking, intra-provincial regional distribution ratio, and specialty composition percentage. The study sorts out the number, regional distribution, specialty layout, and project undertaking characteristics of clinical trial institutions nationwide and in Sichuan Province, with a focus on analyzing the development differences between institutions in Chengdu and non-central cities.

**Results:**

As of September 9, 2025, China had 1,803 registered Good Clinical Practice (GCP) institutions. Sichuan Province ranks the 7th nationwide with 88 registered institutions; however, the distribution within the province is highly uneven (HHI = 0.25, indicating moderate concentration). Chengdu alone accounts for 50.00% of the total (44 institutions), while the coverage rate of institutions in regions such as Western Sichuan and Northeastern Sichuan is less than 30%. Internal medicine dominated the professional distribution (57 institutions, 64.77%), while advanced platforms such as Phase I clinical research units (22 institutions) remained relatively scarce. The number of registered PIs in the province reached 1,424, but only 32.7% were from non-Chengdu institutions. Chengdu Fifth People’s Hospital undertook 203 projects following its record-filing, including 26 bioequivalence (BE) trials.

**Conclusion:**

The record-filing system has substantially expanded clinical trial resources in Sichuan Province. However, persistent challenges include regional disparities, suboptimal specialization structures, and inadequate quality systems. Newly established institutions should proactively address these common issues through high-standard development, thereby contributing to a more balanced, efficient, and high-quality clinical trial ecosystem in the province.

## 1 Introduction

Clinical trials represent a pivotal link in new drug research and development (R&D), with their quality and efficiency directly impacting public health and the development of the pharmaceutical industry (1). Since 2019, the National Medical Products Administration (NMPA) has issued the Regulations on the Administration of Drug Clinical Trial Institutions, formally reforming the qualification certification system for clinical trial institutions into a record-filing management system (2). This landmark policy shift has lowered entry barriers, stimulated market vitality, and encouraged more medical institutions to participate in new drug R&D, thereby addressing the growing demand for clinical trials (3).

Clinical trial institutions serve as core carriers for conducting clinical trials of drugs, medical devices, and biological products. Their capacity and quality directly influence the progress of new drug R&D, the reliability of clinical data, and the protection of subjects’ rights and interests (4, 5). Over the past 5 years, the number of GCP institutions in China has experienced rapid growth. As a major pharmaceutical hub in Western China, the development of GCP institutions in Sichuan Province plays a crucial role in shaping the national pharmaceutical innovation ecosystem (6). However, critical questions remain unanswered: Has the increase in quantity been accompanied by optimized resource structure? Is the allocation of resources across regions and specialties balanced? Currently, there is a lack of systematic analysis based on provincial-level data.

Notably, Yang and Li (7) conducted an analysis of Sichuan’s clinical trial institutions based on data before 2021. Compared with their study, this research has three key innovations: ① Data is updated to September 9, 2025, covering the complete 5-year cycle after the implementation of the record-filing system, enabling a more comprehensive reflection of the policy’s long-term impact; ② A special analysis of BE trials is added to reveal the rising trend of local hospitals in the field of bioequivalence research; ③ Combined with the practice of undertaking 203 projects by our hospital (Chengdu Fifth People’s Hospital), actionable development paths for newly registered institutions are proposed to enhance the practical reference value of the research.

To fill this gap, the present study comprehensively analyzes the development status and existing challenges of GCP institutions nationwide and in Sichuan Province using the latest record-filing data. Combined with national regulatory trends, this study aims to provide evidence-based recommendations for enhancing clinical trial quality, which may inform policy-making and institutional management.

## 2 Methods

### 2.1 Data sources

Primary data were retrieved from two official platforms: ① National Drug Clinical Trial Institution Record-Filing Management Information Platform (8), including basic information of registered institutions (location, grade, specialty registration), PI registration information, and filing time; ② Drug Clinical Trial Registration and Information Disclosure Platform (9), covering project undertaking data (project phase, drug category, BE trial quantity) of institutions nationwide and in Sichuan Province. Secondary data included the Statistical Communique of Tertiary Hospitals in China (2024) issued by the National Health Commission, and regulatory documents such as the Measures for the Daily Supervision and Inspection of Drug Clinical Trial Institutions in Sichuan Province (10).

### 2.2 Data processing and analysis

Data cleaning: Duplicate records, institutions with revoked filing qualifications, and entries missing key information (e.g., location, specialty) were excluded. A total of 1,803 valid national institution records and 88 valid Sichuan institution records were retained.

Key metrics definition (11–13): ① Institution coverage rate = (Number of registered institutions in the region/Total number of tertiary hospitals in the region) × 100%; ② Regional concentration degree: Measured by Herfindahl–Hirschman Index (HHI), calculated as the sum of the squares of the proportion of registered institutions in each prefecture-level city to the provincial total (HHI = 0 indicates complete competition, HHI = 1 indicates complete monopoly); ③ Compound annual growth rate (CAGR) of institutions: Calculated as (Final number/Initial number)^(1/Number of years) – 1.

Analytical methods: Descriptive statistics (frequency, percentage) were used to characterize institution quantity, regional distribution, and specialty composition; trend analysis was employed to explore the growth law of institutions and projects; concentration analysis (HHI) was used to evaluate regional resource agglomeration; inter-provincial and intra-provincial comparative analyses were conducted to identify development gaps.

### 2.3 Data verification

The number of national tertiary hospitals (3,855) was verified with the National Health Commission’s 2024 Statistical Communique; Sichuan’s institutional distribution data was cross-checked with the Sichuan Provincial Medical Products Administration’s supervision records to ensure accuracy.

## 3 National implementation status of the record-filing system for drug clinical trial institutions

### 3.1 Quantity and regional distribution of institutions

**Figure 1:**
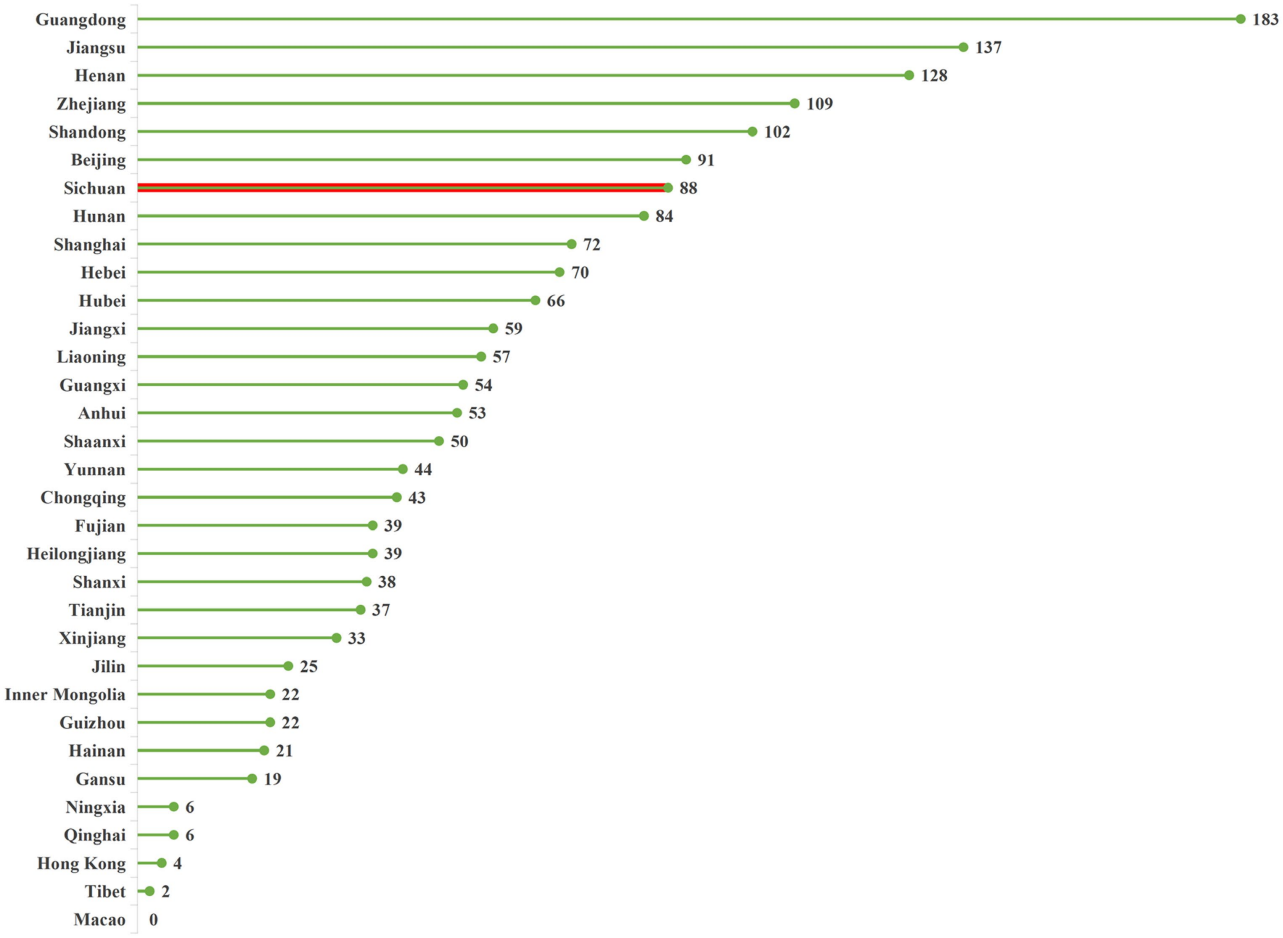
Distribution of the number of registered clinical trial institutions across regions in China. Sichuan Province is highlighted in red. Data source: National Drug Clinical Trial Institution Record-Filing Management Information Platform, 2025. Data cutoff time: September 9, 2025.

According to data from the National Drug Clinical Trial Institution Record-Filing Management Information Platform (8), as of September 9, 2025, the total number of registered institutions nationwide reached 1,803. Geographically, provinces with abundant medical resources maintained a distinct advantage: Guangdong Province (183 institutions), Jiangsu Province (137 institutions), and Henan Province (128 institutions) ranked top three, accounting for 24.8% of the national total. Among Western provinces, Sichuan Province led with 88 institutions and an annual compound growth rate of 18.7% followed by Shaanxi Province (50 institutions) and Chongqing Municipality (43 institutions). Compared with the national total of 3,855 tertiary hospitals, the coverage rate of registered institutions was only 46.8%, with Western provinces recording a mere 10.1% coverage—below the national average. This indicates substantial room for expanding clinical trial resources in Western China. The specific quantity and distribution of registered institutions are presented in Figure 1.

**Figure 2:**
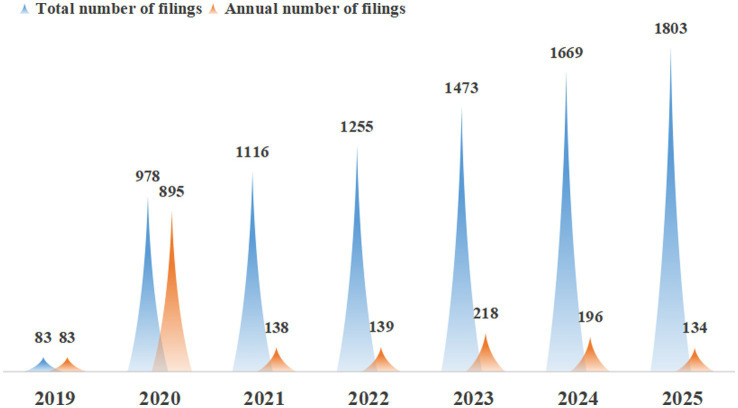
Total number of registered institutions across regions in China and annual number of newly registered institutions. “Total number of filings” refers to the cumulative number of registered institutions by the end of each year; “Annual number of filings” refers to the number of institutions newly registered in the corresponding year. 2019 data only includes institutions registered after the implementation of the record-filing system in December. Data source: National Drug Clinical Trial Institution Record-Filing Management Information Platform. Data cutoff time: September 9, 2025.

Furthermore, data showed that the growth rate of clinical trial institutions accelerated significantly after the implementation of the record-filing system, the annual compound growth rate of national institutions from 2019 to 2025 was 15.3%, with the most notable increase in 2020. By the end of 2020, the number of newly registered drug clinical trial institutions nationwide had reached 978, including 895 institutions that had not obtained prior qualification certification (before December 1, 2019). The annual number of newly registered institutions nationwide is detailed in Figure 2. It should be noted that the number of registered institutions in 2019 was low because the record-filing system was officially implemented in December 2019, and the data only includes institutions registered in that month. This rapid growth reflects the incentive effect of policy liberalization on institutional participation and provides a foundational capacity for undertaking more clinical trial projects.

### 3.2 Project undertaking by national institutions

**Figure 3:**
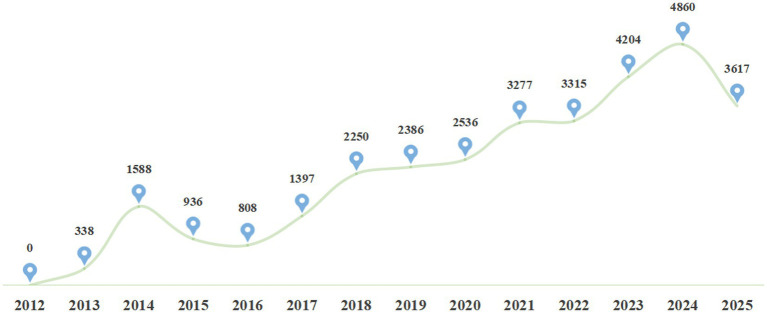
Annual number of clinical trial projects undertaken by clinical trial institutions nationwide. 2025 data includes only projects registered before September 9. Data source: Drug Clinical Trial Registration and Information Disclosure Platform. Data cutoff time: September 9, 2025.

As of September 9, 2025, a total of 31,515 trials had been registered on the Drug Clinical Trial Registration and Information Disclosure Platform (9). Detailed information is presented in Figure 3. Among these, domestic trials accounted for 91.87%, international multi-center trials (MRCTs) for 7.84%, and others for 0.29%. By drug category, chemical drugs constituted 75.02%, traditional Chinese medicine (TCM)/natural drugs 3.88%, and biological products 21.1%. Results indicated that since the reform of the record-filing system in 2019, the number of clinical trial projects has increased in tandem with the growth of registered institutions, peaking at 4,860 registered projects in 2024. The “slight decline” in 2025 is due to the data cutoff time (September 9), which only includes the first three quarters of 2025; the total number of projects for the full year is expected to be comparable to or slightly higher than that in 2024.

## 4 Development status of clinical trial institutions in Sichuan Province

### 4.1 Quantity and distribution characteristics

**Figure 4:**
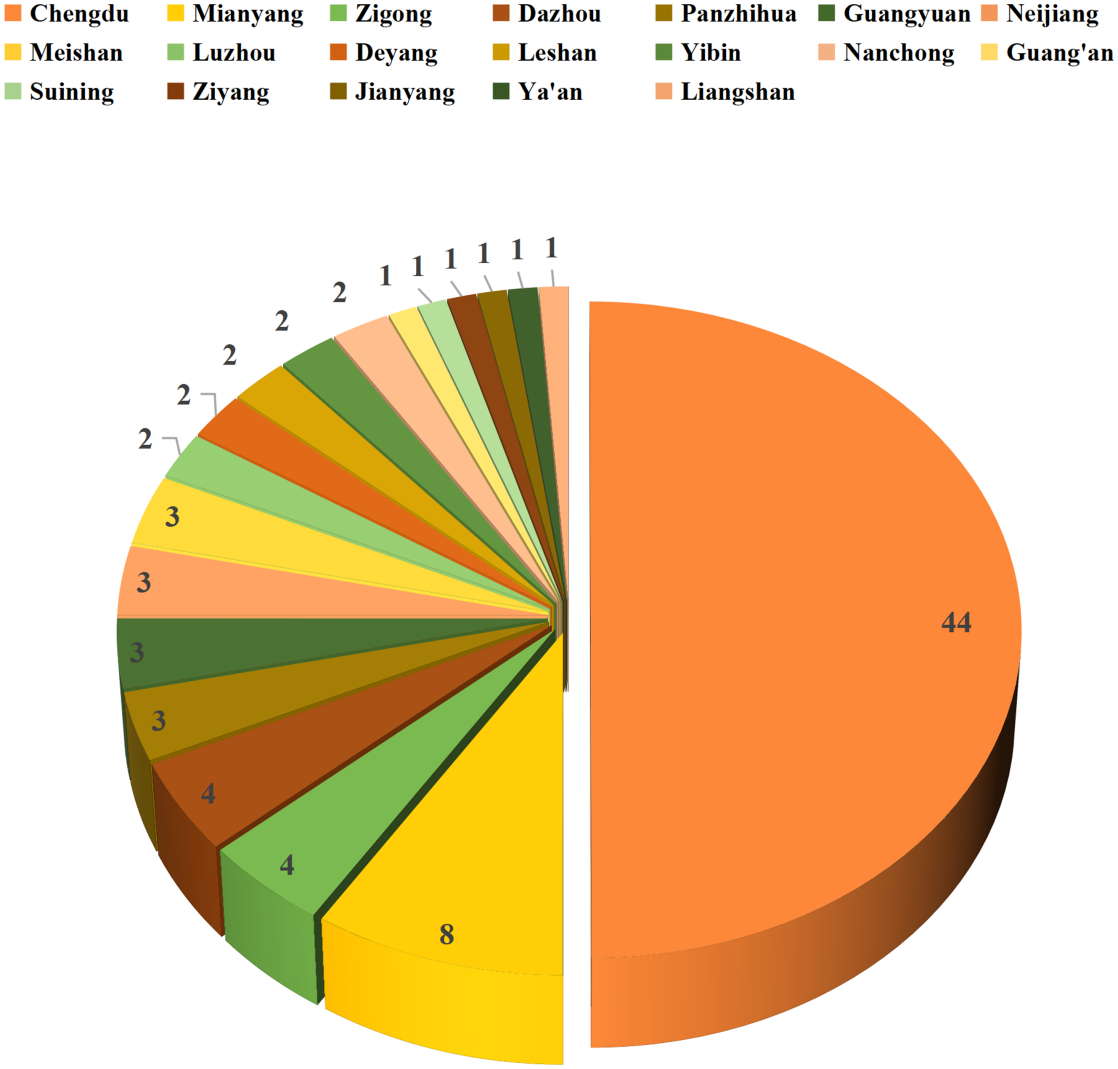
Distribution map of clinical trial institutions in Sichuan Province. The numerical values in the figure represent the number of registered institutions in each prefecture-level city/autonomous prefecture. Chengdu is highlighted in orange. Data source: National Drug Clinical Trial Institution Record-Filing Management Information Platform. Data cutoff time: September 9, 2025.

Sichuan Province’s 88 registered institutions are distributed across 18 prefecture-level cities and autonomous prefectures, excluding Bazhong, Garzê Tibetan Autonomous Prefecture, and Aba Tibetan and Qiang Autonomous Prefecture. While geographically widespread, the distribution is highly concentrated (HHI = 0.25, indicating moderate concentration, Figure 4). Chengdu, as the provincial capital, hosts 44 registered institutions—accounting for 50% of the provincial total—exhibiting a strong resource agglomeration effect.

This distribution pattern is closely linked to multiple factors: ① Economic development disparities: The Chengdu Plain economic circle has a per capita GDP 2.3 times that of Western Sichuan and Northeastern Sichuan, supporting higher investment in clinical trial infrastructure; ② Historical layout of high-quality medical resources: 70% of Sichuan’s top tertiary hospitals are concentrated in Chengdu, providing a solid foundation for clinical trial development; ③ Policy incentives: Early pilot policies for the record-filing system were primarily implemented in core cities, further attracting resource agglomeration (14–17). This imbalance limits access to clinical trial participation for patients in some areas, particularly hindering research on local diseases and ethnic minority-specific conditions (18).

### 4.2 Registered specialties and principal investigator (PI) status

**Figure 5:**
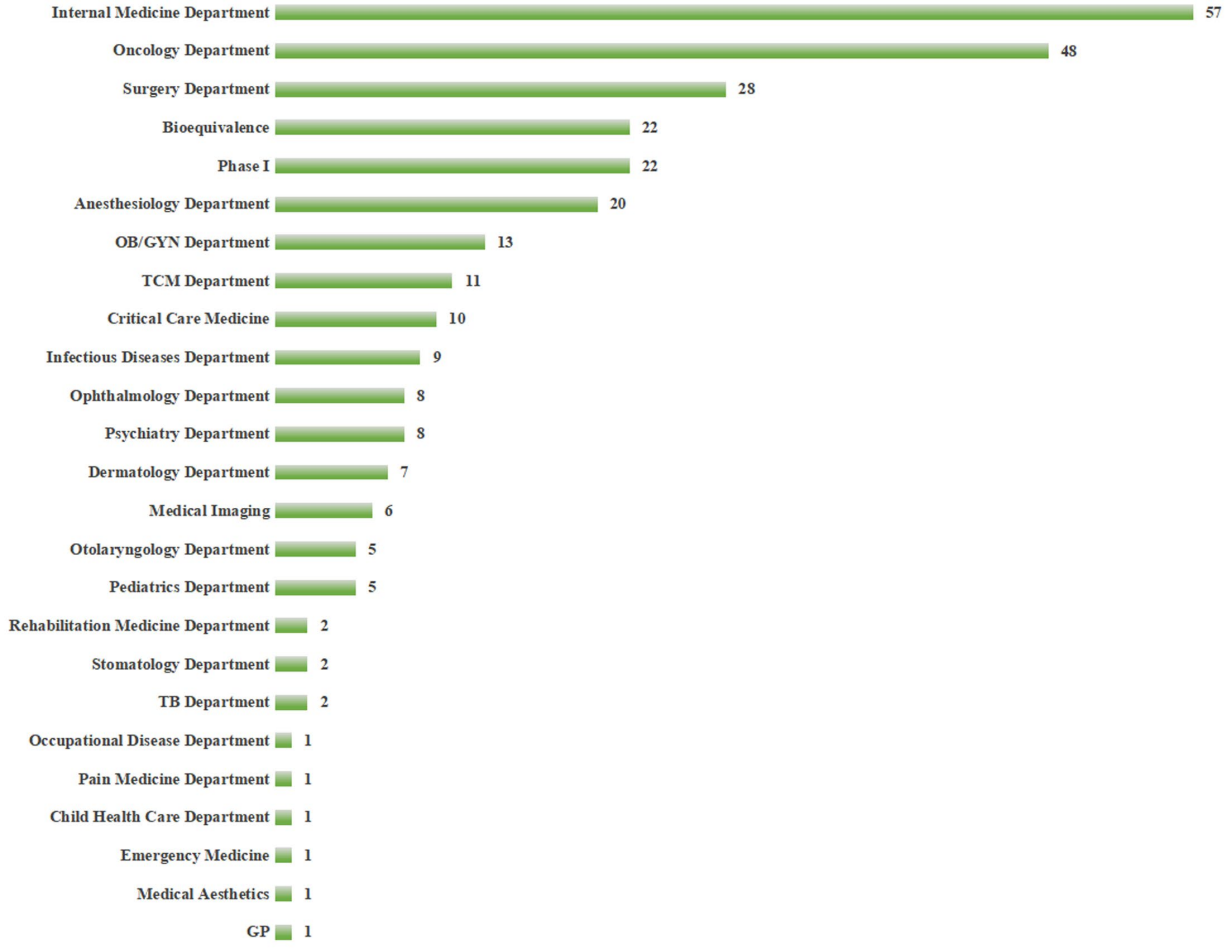
Distribution chart of registered specialties of drug clinical trial institutions in Sichuan Province. General Practice = GP; Tuberculosis Department = TB Department; Traditional Chinese Medicine Department = TCM Department; Obstetrics and Gynecology Department = OB/GYN Department; “Internal medicine” includes cardiovascular, gastroenterology, respiratory, and other subspecialties permitted by NMPA. Data source: Sichuan Provincial Medical Products Administration. Data cutoff time: September 9, 2025.

The distribution of registered specialties in Sichuan Province is shown in Figure 5. Internal medicine is the most commonly registered specialty, with 57 institutions (64.77%), consistent with its broad disease spectrum and large patient base. It should be clarified that “Internal medicine” is a core specialty category permitted by the NMPA’s record-filing system, covering subspecialties such as cardiovascular medicine, gastroenterology, and respiratory medicine. Additionally, 22 institutions in the province have registered Phase I clinical research units—high-standard platforms that require advanced hardware and software facilities, qualified researchers, and robust management systems. These units are typically concentrated in top-tier tertiary hospitals (e.g., West China Hospital, Sichuan University) and serve as core indicators of a region’s original innovation capacity (19).

A total of 1,424 PIs have completed registration in the province, who are the primary safeguards for clinical trial quality (5). Notably, PI qualifications are a key factor for successful institutional registration and high-quality trial conduct (20). However, data shows that only 32.7% of registered PIs are from non-Chengdu institutions, and nearly 40% of newly registered institutions have PIs lacking experience in Phase I or MRCT projects, which directly restricts their ability to undertake complex trials.

### 4.3 Project undertaking status

**Figure 6:**
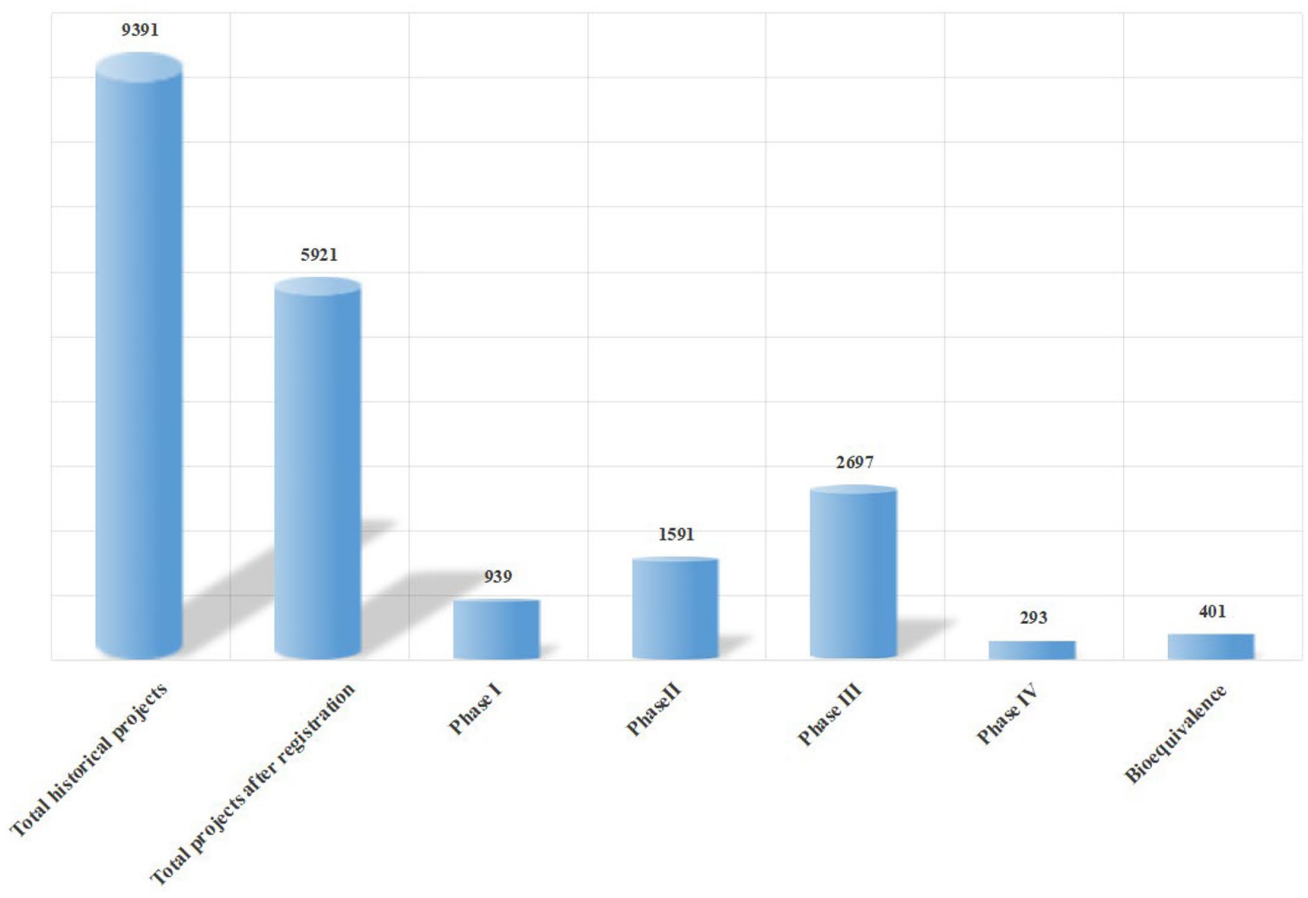
Summary of clinical trial projects undertaken province-wide and their phases. “Total historical projects” refers to the cumulative number of projects undertaken by provincial institutions since their establishment; “Total projects after registration” refers to the number of projects undertaken after the implementation of the record-filing system. Data source: Drug Clinical Trial Registration and Information Disclosure Platform. Data cutoff time: September 9, 2025.

As of September 9, 2025, statistical data on project undertaking in Sichuan Province showed a total of 9,391 historically archived projects, including 401 BE trials. During the current statistical period, 5,921 new projects have been undertaken since institution registration. Figure 6 illustrates the distribution of projects undertaken across the province, revealing significant differences in the number of projects across phases: Phase III projects accounted for the highest proportion, while Phase IV projects were relatively scarce. This discrepancy may reflect inefficiencies in phase transition or suboptimal resource allocation, providing valuable insights for regional research management and policy formulation (21).

Among the top 11 institutions in Sichuan Province by the total number of historical clinical trial projects undertaken, West China Hospital, Sichuan University ranked first with 3,423 projects—far exceeding other institutions. Sichuan Provincial People’s Hospital (1,136 projects) and Sichuan Cancer Hospital (837 projects) ranked second and third, respectively, while the remaining institutions undertook fewer than 500 projects each.

**Figure 7:**
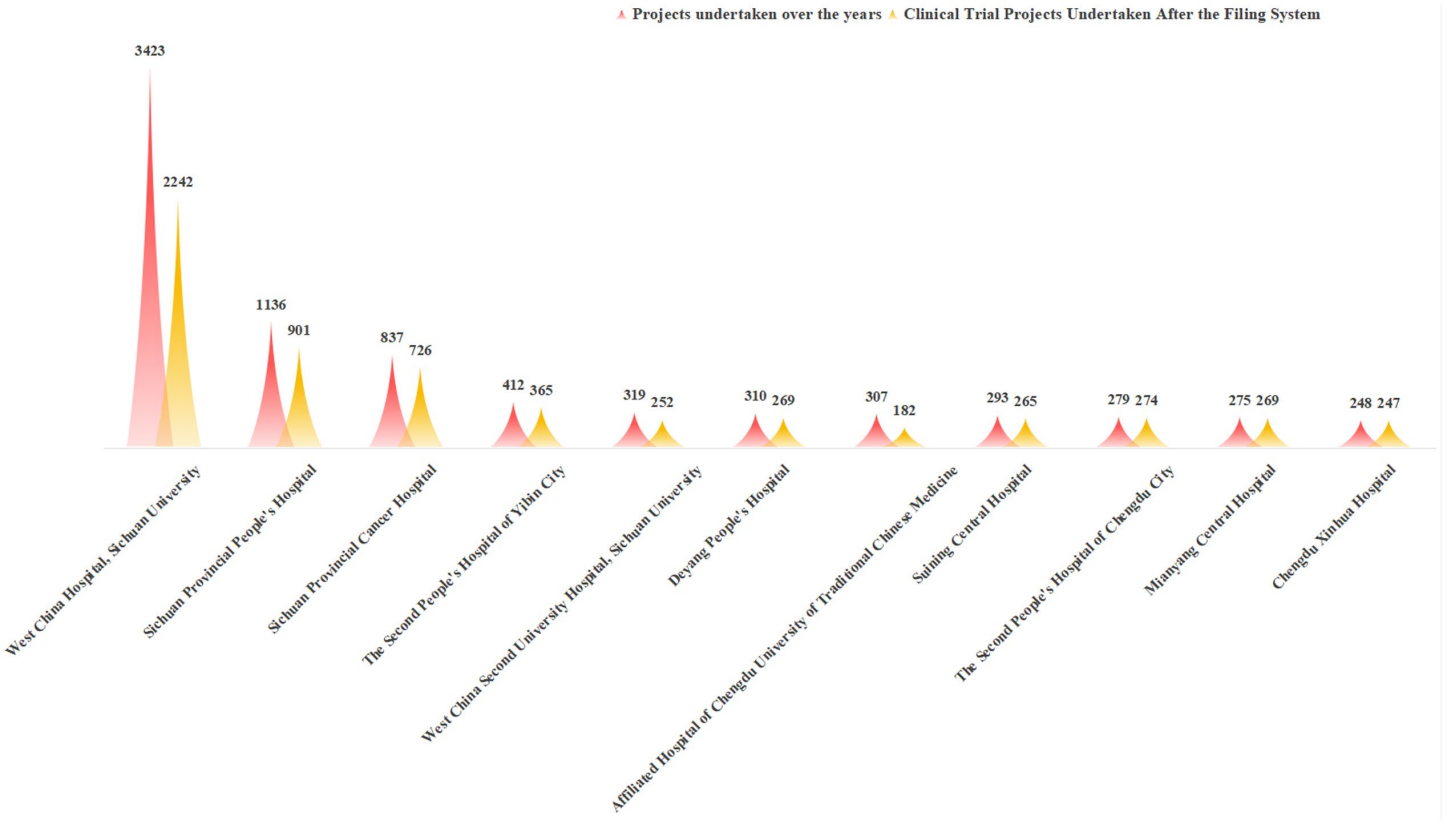
Distribution of the top 11 institutions in undertaking clinical trial projects before and after the filing system. Data source: Drug Clinical Trial Registration and Information Disclosure Platform, 2025.

Since the implementation of the record-filing system in December 2019, West China Hospital has maintained its leading position with 2,242 projects, followed by Sichuan Provincial People’s Hospital (901 projects) and Sichuan Cancer Hospital (726 projects). Most other institutions undertook between 200 and 300 projects during this period. Notably, the Affiliated Hospital of Chengdu University of TCM has dropped out of the national top 10 in terms of project quantity after the record-filing system, while Chengdu Xinhua Hospital has achieved significant growth in project undertaking, performing exceptionally well. The majority of other hospitals have maintained stable rankings among the top 10 in both historical and post-registration project undertaking, with specific comparisons shown in Figure 7.

**Figure 8:**
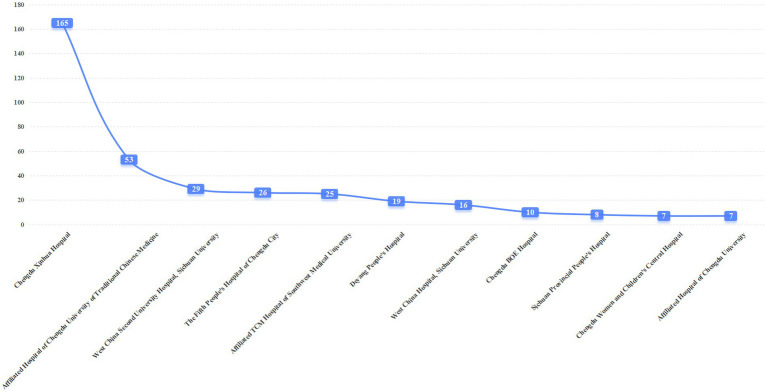
Distribution of the top 11 institutions in undertaking BE projects after the filing system. Data source: Drug Clinical Trial Registration and Information Disclosure Platform, 2025.

As of September 9, 2025, the undertaking of BE trials has undergone significant changes. Chengdu Fifth People’s Hospital, Affiliated TCM Hospital of Southwest Medical University, Chengdu BOE Hospital, Chengdu Women and Children’s Central Hospital, and Affiliated Hospital of Chengdu University have entered the top 10 in BE trial quantity for the first time. Among them, Chengdu Xinhua Hospital ranked first with 165 BE trials, followed by the Affiliated Hospital of Chengdu University of TCM (53 trials) and West China Second University Hospital, Sichuan University (29 trials). Our hospital (Chengdu Fifth People’s Hospital) undertook 26 BE trials, ranking fourth. Detailed project information is presented in Figure 8.

Overall, West China Hospital continues to lead in total project undertaking but does not dominate the BE trial sector, reflecting distinct professional division of labor among institutions in different types of clinical trials (22). After the implementation of the record-filing system, the overall pattern of project-undertaking institutions remains stable; however, some local hospitals have performed prominently in BE research, which may be attributed to their research positioning and resource allocation strategies.

### 4.4 Special features of Sichuan’s institutional analysis

Compared with similar analyses in other provinces, Sichuan’s clinical trial institution development has distinct characteristics (23, 24): ① As a western medical hub, it undertakes a large number of cross-regional clinical trial projects from western provinces; ② Local hospitals have risen rapidly in BE trials, breaking the long-term monopoly of top-tier hospitals, forming a “dual-track development” pattern of “top hospitals undertaking complex MRCTs + local hospitals focusing on BE trials”; ③ There is an urgent demand for clinical trials of ethnic medicines (e.g., Tibetan medicine, Qiang medicine) in ethnic minority areas, which provides a unique direction for the future layout of institutions.

## 5 Discussion

### 5.1 Summary of key findings

This study’s core findings include: ① After the implementation of the record-filing system, the number of drug clinical trial institutions nationwide and in Sichuan Province has grown rapidly, with Sichuan ranking first in Western China with 88 institutions and an annual compound growth rate of 18.7% (higher than the national average of 15.3%); ② Regional distribution is moderately concentrated (HHI = 0.25), with Chengdu accounting for 50% of institutions, and the coverage rate in remote areas is less than 30%; ③ The specialty layout is dominated by internal medicine, with Phase I research platforms being scarce (only 22 institutions); ④ In terms of project undertaking, top-tier hospitals lead in total quantity, while local hospitals have emerged as important forces in BE trials; ⑤ PI qualification gaps and uneven quality management systems are key challenges for newly registered institutions.

### 5.2 Challenges under the record-filing system and regulatory trends

While the record-filing system has simplified access, it has also increased regulatory complexity and quality risks. Since 2024, summaries of rectification issues for clinical trial institutions released by multiple provinces and municipalities nationwide (including Tianjin, Hebei, Shanghai, Jiangsu, and Zhejiang) have revealed common challenges (25–27). Inadequate qualifications and capabilities of PIs and researchers are a top priority in inspections, with key issues including PIs’ inability to provide evidence of participating in at least 3 registration-oriented trials, weak GCP awareness among researchers, poor protocol compliance, inadequate training, and unstable research teams. Ineffective operation of quality assurance systems is another prominent problem, as internal quality control (QC) within institutions is often a mere formality that fails to effectively identify and correct deviations during trials, while ethics committees lack sufficient review capacity and conduct inadequate follow-up reviews. Questionable data authenticity and traceability also pose significant risks, with source data recorded untimely and non-standardly, inconsistencies between Case Report Forms (CRFs) and source data, and untraceable original records such as medical charts. Additionally, non-standard management of investigational products—including lack of temperature monitoring and counting errors—and insufficient protection of subjects’ rights and interests (e.g., non-standard informed consent processes) are common issues.

The exposure of these challenges indicates that regulatory focus has shifted from “qualification access” to “process supervision” and “continuous capacity assessment” (28). In response, the Sichuan Provincial Medical Products Administration has established a full-cycle regulatory model for institutions, including mandatory inspections within 60 working days for newly registered institutions, full-coverage inspections every 3 years, and “follow-up inspections” for institutions with identified issues (10). Notably, PI qualifications are the core key to the success of institutional registration and trial conduct; for new institutions, recruiting PIs with sufficient project experience is extremely difficult (20), a bottleneck that is particularly prominent in non-central cities of Sichuan and restricts the development of local clinical trial institutions.

### 5.3 Countermeasures and recommendations

To promote the healthy development of the clinical trial ecosystem in Sichuan Province, the following targeted recommendations are proposed (29–32):

#### 5.3.1 Optimize regional layout and strengthen differentiated guidance

Provincial health commissions and drug regulatory authorities should formulate a “1 + N” regional collaborative development plan, taking Chengdu’s top institutions as “hubs” to establish regional sub-centers in Mianyang, Luzhou, and Yibin. Targeted policy and technical support should be provided for remote areas, including organizing centralized GCP training (no less than 4 times a year) for researchers in non-central cities, establishing a remote mentoring mechanism between top institutions and grassroots institutions with senior PIs assigned to provide one-on-one guidance, and setting up special funds to support the purchase of EDC systems and temperature monitoring equipment for institutions in remote areas.

#### 5.3.2 Focus on PI team cultivation and improve talent echelon

Institutions should implement strict PI access control by verifying qualifications through multiple channels (e.g., project approval documents, research reports) to ensure compliance with NMPA requirements. A hierarchical training system should be established: for senior PIs, ICH GCP advanced training should be conducted to enhance MRCT undertaking capabilities; for young researchers, a “PI reserve talent program” should be launched to provide systematic training in trial design, data management, and ethical review. Incentive mechanisms should also be improved by including clinical trial achievements in physician promotion and performance evaluation indicators, and increasing salary subsidies for CRCs to reduce turnover.

#### 5.3.3 Consolidate quality management and standardize full-process control

All institutions should establish a three-tier quality control system (investigator self-inspection, departmental inspection, institutional inspection) with monthly self-inspection reports and quarterly summary analyses. The construction of ethics committees should be strengthened by recruiting legal, statistical, and community representatives, and conducting special training on the review of vulnerable subjects and emergency scenarios. Digital management should be promoted by encouraging institutions to adopt CTMS and EDC systems, and realizing interconnection with HIS and LIS systems to ensure real-time, accurate, and traceable data.

#### 5.3.4 Guide newly registered institutions to establish clear positioning

Newly registered institutions should avoid blind competition and focus on differentiated development: concentrate resources on 2–3 specialties with strong clinical capabilities to form core competitiveness; cooperate with experienced CROs and top institutions to accumulate experience through project participation; and seize the BE trial market as an entry point to gradually improve management and technical capabilities (as demonstrated by Chengdu Fifth People’s Hospital’s experience of undertaking 26 BE trials).

### 5.4 Limitations

This study has several limitations that need to be acknowledged: ① Single-province case study: Sichuan’s status as a western medical hub may limit the direct applicability of findings to other provinces with different economic and medical resource bases (e.g., eastern coastal provinces or underdeveloped western provinces); ② Cross-sectional design: The study uses data up to September 2025, which cannot fully reflect the dynamic changes in the record-filing system’s impact over time (e.g., long-term quality improvement of newly registered institutions); ③ Lack of multi-stakeholder perspectives: The analysis primarily focuses on institutional and regulatory viewpoints, lacking insights from sponsors, CROs, and trial participants, which may lead to incomplete understanding of practical challenges; ④ Limited analytical depth: Although concentration analysis (HHI) is added, more advanced statistical methods (e.g., regression analysis) are not used to explore the impact of economic development level on institutional distribution, which can be supplemented in future research.

## 6 Conclusion

The implementation of the record-filing system represents a profound reform in China’s drug clinical trial field, significantly expanding the supply of clinical trial resources in Sichuan Province. However, quantitative growth is not an end in itself; achieving a transformation from “quantity” to “quality” and from “existence” to “excellence” is the core challenge currently facing the sector. Newly established institutions should learn from common national issues, adhere to high standards and strict requirements, focus on advantages, and make steady progress. Only in this way can they truly seize the historical opportunity brought by the record-filing system and contribute to improving the overall level of pharmaceutical R&D in the province and the country.

## Data Availability

The original contributions presented in the study are included in the article/supplementary material, further inquiries can be directed to the corresponding author.
